# An *in vivo* comparison study in goats for a novel motion-preserving cervical joint system

**DOI:** 10.1371/journal.pone.0178775

**Published:** 2017-06-05

**Authors:** Jie Qin, Chenguang Zhao, Dong Wang, Bo Zhao, Jun Dong, Haopeng Li, Rongxia Sang, Shuang Wang, Jiao Fu, Rangrang Kong, Xijing He

**Affiliations:** 1 The Department of Orthopedics, the Second Affiliated Hospital of Xi'an Jiaotong University, Xi'an, Shaanxi Province, P. R. of China; 2 The Department of Rehabilitation, Xijing Hospital, Xi'an, Shaanxi Province, P.R. of China; 3 The Department of Gastroenterology, the First Hospital of Shijiazhuang, Shijiazhuang, Hebei Province, P.R. of China; 4 Institute of Photonics and Photon-technology, Northwest University, Xi’an, Shaanxi Province, P.R. of China; 5 The Department of Endocrinology, the First Affiliated Hospital of Xi'an Jiaotong University, Xi'an, Shaanxi Province, P.R. of China; 6 The Department of Thoracic Surgery, the Second Affiliated Hospital of Xi'an Jiaotong University, Xi'an, Shaanxi Province, P.R. of China; University of Toronto, CANADA

## Abstract

Cervical degenerative disease is one of the most common spinal disorders worldwide, especially in older people. Anterior cervical corpectomy and fusion (ACCF) is a useful method for the surgical treatment of multi-level cervical degenerative disease. Anterior cervical disc replacement (ACDR) is considered as an alternative surgical method. However, both methods have drawbacks, particularly the neck motion decrease observed after arthrodesis, and arthroplasty should only be performed on patients presenting with cervical disc disease but without any vertebral body disease. Therefore, we designed a non-fusion cervical joint system, namely an artificial cervical vertebra and intervertebral complex (ACVC), to provide a novel treatment for multi-level cervical degenerative disease. To enhance the long-term stability of ACVC, we applied a hydroxyapatite (HA) biocoating on the surface of the artificial joint. Thirty-two goats were randomly divided into four groups: a sham control group, an ACVC group, an ACVC-HA group, and an ACCF group (titanium and plate fixation group). We performed the prosthesis implantation in our previously established goat model. We compared the clinical, radiological, biomechanical, and histological outcomes among these four different groups for 24 weeks post surgery. The goats successfully tolerated the entire experimental procedure. The kinematics data for the ACVC and ACVC-HA groups were similar. The range of motion (ROM) in adjacent level increased after ACCF but was not altered after ACVC or ACVC-HA implantation. Compared with the control group, no significant difference was found in ROM and neutral zone (NZ) in flexion-extension or lateral bending for the ACVC and ACVC-HA groups, whereas the ROM and NZ in rotation were significantly greater. Compared with the ACCF group, the ROM and NZ significantly increased in all directions. Overall, stiffness was significantly decreased in the ACVC and ACVC-HA groups compared with the control group and the ACCF group. Similar results were found after a fatigue test of 5,000 repetitions of axial rotation. The histological results showed more new bone formation and better bone implant contact in the ACVC-HA group than the ACVC group. Goat is an excellent animal model for cervical spine biomechanical study. Compared with the intact state and the ACCF group, ACVC could provide immediate stability and preserve segmental movement after discectomy and corpectomy. Besides, HA biocoating provide a better bone ingrowth, which is essential for long-term stability. In conclusion, ACVC-HA brings new insight to treat cervical degenerative disease.

## Introduction

Cervical degenerative disease is a prevalent problem in our aging population. It can be in the form of herniated discs and spondylosis in the cervical spine associated with axial neck and/or radicular arm pain and neurological symptoms. Surgical and nonsurgical treatments have been used to halt or reverse the disease process. Posterior approachesas well as anterior techniques, such as anterior cervical discectomy and fusion (ACDF) and anterior cervical corpectomy and fusion (ACCF), are the most commonly used approaches.

Because of its high rate of clinical success (relief of symptoms and favorable outcomes),anterior cervical discectomy and fusionhas been considered by many orthopedist and neurosurgeons as the standard surgical treatment for cervical myelopathy or radiculopathy, secondary to cervical degenerative disease and refractory to a conservative treatment[1]. The anterior cervical discectomy or subtotal corpectomy directly remove the compression induced, for example, by the protrude disc, and also permanently eliminate the pathogenic factor by fusing the degenerative segments[2, 3]. However, ACCF has some limitations that include graft donor site morbidity, subsidence, debris, pseudarthrosis, and adjacent segment disease. Adjacent segment cervical disease occurs in approximately 3% of patients per year, with an expected incidence of 25% within the first 10 years following fusion[4]. Goffinet al.[5] found that after anterior cervical fusion, 92% of patients displayed additional radiological adjacent segmental degeneration, from which 25% presented with clinical adjacent segmental disease in a 10-year follow-up. Matsumotoet al.[6] performed a prospective 10-year follow-up magnetic resonance imaging (MRI) study on 64 patients who underwent anterior cervical discectomy and fusion and 201 asymptomatic healthy control subjects. They demonstrated that although both groups showed a progression towards disc degeneration during the 10-year period, patients treated with anterior cervical fusion had a significantly higher incidence of progression towards disc degeneration at adjacent segments than controls, even if this was not always related to the development of clinical symptoms. Besides, although the relationship between adjacent segmental disease and anterior cervical fusion remains unanswered, the motion loss following ACCF results in an increased range of motion and a higher intradiscal pressure in the facet joint adjacent to the fusion level, which is considered to be related to the adjacent segmental disease [7]. This concern has led to the development of motion-preserving devices such as artificial cervical discs.

Artificial cervical disc replacement (ACDR) has developed and is now increasingly used as an alternative to ACCF. The first clinical trial involving spinal arthroplasty occurred between 1962 and 1970 and implied the implantation of Fernstrom ball in 207 cases. Nowadays, some clinical trials are currently testing various artificial cervical disc devices while some have been approved by the United States Food and Drug Administration. Arthroplasty seems to be as safe as ACCF and is becoming a substitute for fusion. Cepoiu-Martin et al.[8] performed a systematic review and reported that ACDR appeared to be at least as effective as ACDF in the short-term follow-up (up to 2 years). The theoretical advantages of ACDR are the restoration of the intervertebral disc and the prevention of nerve root compression recurrence. The preservation of physiological spine mobility, rather than the fusion of the degenerative spine, may limit the progression of adjacent segmental degeneration. However, ACDR has some drawbacks, such as more strict criteria about the intervertebral disc and the vertebral body than ACCF.

For multi-level cervical degeneration disease, there is currently no method to rebuild cervical stability and preserve the motion function after decompression. There is no role for the indiscriminate use of ACDR. ACDR should simply not be considered as superior or as a replacement of fusion. Besides, if there is a vertebral body disease, such as a primary tumor, metastatic cancer, tuberculosis, vertebral body deformity, fusion is still the most commonly used surgical technique. To address these issues, we developed a novel artificial cervical vertebra and intervertebral complex (ACVC) that can provide immediate stability after anterior cervical corpectomy and maintain the motion of the surgery segment. Theoretically, this motion-preserving joint system may prevent adjacent segment degeneration.

Moreover, long-term stability of prostheses is necessary for orthopedic implants. For uncemented prostheses, many parameters could affect long-term stability, including the implant design, surface texture, material biocompatibility, the presence of a bioactive coating, and the biomechanical properties of the surrounding bone. Various biocoatings have been proposed as effective methods for improving the bone-implant interface stability. In recent decades, hydroxyapatite ((Ca_10_(PO_4_)_6_(OH)_2_; HA) has been reported to have a high biocompatibility, a low degradation rate, and the ability to increase the efficiency of bone-implant integration in order to improve osteointegration of implants because of its similar composition to bone tissue and its ability tocreate chemical bonding withbones[9]. Xie and Luan[10] reported that the precoated HA could further improve the stability of the Ti6Al4V alloy with a composite layer of TiO_2_ and HA. This suggests that artificial joints pretreated with an HA coating may induce strong bonding with the bone via the natural growth of new HA. For this reason, we applied HA biocoatings to the surface of the ACVCs to determine whether the HA biocoating would increase the implant’s interaction with bone and improve its long-term stability.

## Materials and methods

### Study design

In our previous study [11], we found that goat was an appropriate animal model for the biomechanical study of the cervical spine. Specifically, we showed that the C2-C4 vertebrae were the best segments for obtaining biomechanical data comparable to the human cervical spine. We successfully designed the ACVC and implemented an anterior cervical corpectomy and ACVC replacement in goat model. However, some concerns remained, notably regarding the potential advantages of using ACVC over ACCF. Does ACVC result in less kinematic changes compared with ACCF, which is the traditional surgery for cervical degeneration disease? Moreover, how is the long-term stability of ACVC compared with ACCF? To solve these issues, we developed a titanium mesh cage and an anterior cervical plate system for the goat to implement the ACCF animal model, and the ACVC-HA(ACVC with an HA biocoating). In the present study, we compared the clinical features and the biomechanical properties of the ACVC and ACVC-HA with those of the intact state and of the titanium cage and the anterior plate fusion state in goats. Thirty-two goats were randomly divided into four groups and were followed-up for 24 weeks after surgery. Clinical evaluation, radiological observation, biomechanical testing, and histology staining were performed. This study was carried out in strict accordance with the recommendations in the Guide for the Care and Use of Laboratory Animals of the National Institutes of Health. The protocol was approved by the Animal Care and Use Committee of Xi'an Jiaotong University Second Affiliated Hospital approved protocol (Permit Number: 2013–16). All surgeries were performed under anesthesia, and all efforts were made to minimize suffering during the surgical procedures and post-surgery recovery.

### Internal instruments and animal grouping

Thirty-two adult Chinease White goats (age: 23±5 months; average body weight: 57.4±6.7 kg; equal numbers of female and male goats) from the Animal center of Xi'an Jiaotong University Second Affiliated Hospital were randomly divided into 4groups. This included: a control group (Group A), an ACVC group (Group B), an ACVC-HA group (Group C), and an ACCF group (Group D). In the control group, each goat received a sham anterior cervical operation that fully exposed the anterior vertebral surface without disturbing the bony, disc and ligamentous structures. In the ACVC and ACVC-HA groups, ACVC and ACVC-HA were separately implanted in the goats after decompression. In the ACCF group (decompression and fusion group), each animal received an anterior cervical decompression and fusion, using a titanium mesh cage and an anterior plate. To exclude cervical spine abnormalities, radiological images were obtained using a C-arm fluoroscopic imager before surgery.

**Fig 1:**
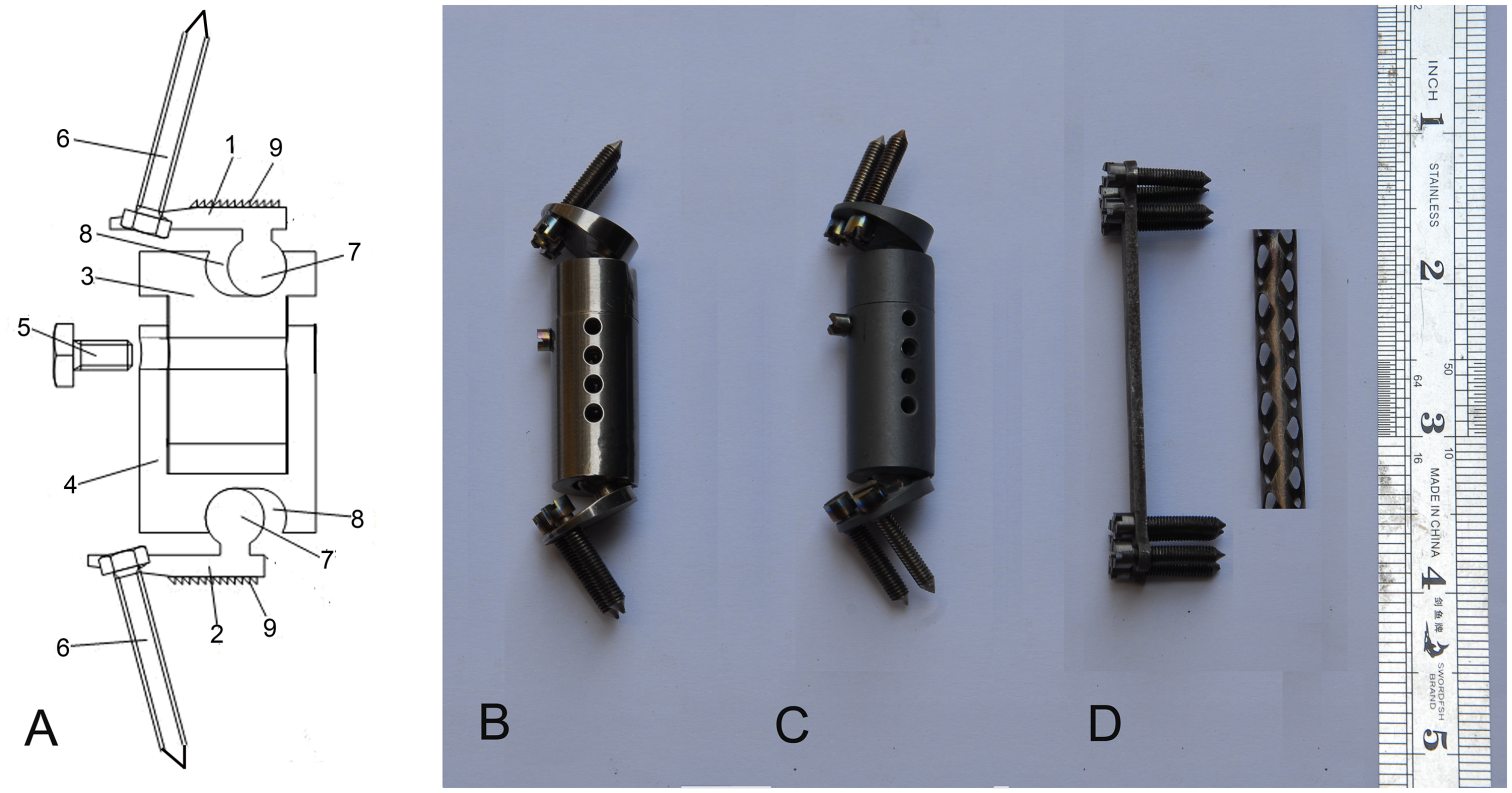
Scheme diagram and photos of the three different artificial implants. A, Scheme diagram of the ACVC; B, actual photo of the ACVC; C, actual photo of the ACVC-HA; D, actual photo of the titanium cage, anterior plate, and fixing screws.

All the internal instruments were made by professional engineers in Northwest Nonferrous Metal Research Institute (China) and were composed of titanium alloy (Ti6Al4V). The ACVC (Patent NO.: US9452062B2; Fig 1B) consisted of two endplate components, two vertebral components, one length-locking screw, and four self-drilling trapping screws. The dimensions of this artificial joint were based on the anatomical and radiological data from the goats. The ACVC is fixed using four self-drilling trapping screws. To create the ACVC-HA (Fig 1C), an HA biocoating with a 30-μm thickness was applied to the surface of the vertebral components, the upper surface of the upper endplate component, the lower surface of the lower endplate component, and the surface of the self-drilling trapping screws, using microarc oxidation (MAO) technology. The biocoating may induce more bone ingrowth, which would facilitate long-term stability.

The design of the titanium cage and the anterior plate (Fig 1D) used in the present study were based on the anatomical data of goat cervical spines and were inspired by the titanium cage and the anterior plate used in human ACCF. Autologous bone could be placed within the hollow structure of the titanium cage. Four to eight self-drilling trapping screws could fix the plate into the adjacent vertebral bodies.

### Artificial cervical joint implantations and postoperative observations

The goats were allowed one-week acclimatisation before the surgery and were housed individually in a thermo-hygrostat cage (23°C, 45%–50% humidity) with 12-hours dark-light cycle. Each goat was given access to food and water *ad libitum*.

The surgical procedures and postoperative animal care have been well documented in our previous study[11]. Briefly, all the animals were given 0.04 mg/kg of atropine subcutaneously, followed by 10 mg/kg of ketamine and 0.3 mg/kg of xylazine intramuscularly. 6 mg/kg of 2.5% thiamylal sodium solution was administered intravenously after 10 minutes. General anesthesia was maintained with approximately 1% halothane in O_2_ through 8-mm diameter endotracheal tube. Then the surgeries were performed in supine position by a transverse skin incision at the C3 level from the right side[12]. The anterior surface of C2-C4, between the carotid sheath and tracheal esophageal sheath, was exposed. At this point, the goats of the control group were sutured. All the other goats underwent anterior C2/C3 and C3/C4 discectomies, and a C3 subtotal corpectomy. Great care was taken to remove the cartilaginous tissue, while preserving intact endplates. After removal of the interbody discs and partial C3 vertebral body, preparation of the endplates with arongeur and a curette, the space left free by the intervertebral discs and the C3 vertebral body was filled with the corresponding implant under anterior distraction. Thus, the goats underwent either ACVC implantation, ACVC-HA implantation or titanium mesh cage and anterior plate implantation according to their group assignments. Final X-ray films were taken to verify the placement of implants before the animals were sutured in layers. Then, the goats were placed in plaster neck casts for 4 weeks to restrict neck activity and facilitate fixation of the implant.

Surgery time, blood loss, recovery time, and time before eating were recorded. Surgery time was the period from skin incision to skin suture. Blood loss was estimated using a vacuum extractor and blood gauze. Recovery time was the period from the end of surgery until the goat could stand by itself. Time before eating was the period from the completion of surgery until the goat began to eat by itself. Food and water intake was limited during the first 24 hours after surgery to reduce the risk of intestinal tympanites. A normal diet was established two days after surgery. Cefazolin (25 mg/kg) was administered twice a day for two days after surgery. Goat care was ensured by trained animal care staff. Eating habits, ambulatory activities, health status, and neurological functions were monitored daily for the first 4 weeks, twice weekly for Weeks 5 to 12, and weekly from the 12th to 24th week.

Post-surgery pain was the major suffering during the post-surgery recovery. Analgesia or anesthesia was used to relieve the post-surgery pain. NSAIDS (non-steroidal anti-inflammatory drugs, such as ibuprofen) and opioids (such as meperidine and morphine) were used to relieve the post-surgery pain if necessary. Soft food (such as cabbages) was provided within the first week after surgery.

Anteroposterior and lateral radiographic films were taken under general anesthesia at weeks 1, 6, 12, and 24 after surgery. Computed tomography (CT) analyses were performed to determine the extent of bony fusion and the position of the prosthesis at weeks 6, 12 and 24 post-surgery. All animals were observed for 24 weeks before euthanasia with an overdose of pentobarbital (200 mg/kg).

Criteria for early euthanasia during the experiment for humane endpoints: weight loss of 20–25%, inappetance up to 5–7 days, severely weakness, moribund state, infection fails to respond to antibiotic therapy, and severe organ system dysfunction non-responsive to treatment.

### Biomechanical tests

The biomechanical test protocol has been described previously [11]. To investigate the adjacent level movement, the C1-C7 segments were dissected from the harvested cervical spines immediately after sacrifice and cleaned of residual soft tissue, with care taken to not disturb the spinal bony and ligamentous attachments. The C1-C7 vertebrae segments were embedded in a special metal mould containing polymethyl-methacrylate to keep the specimens in a set position with the C3 vertebra parallel to the horizontal plan. Three-dimensional flexibility tests were conducted on each of the specimens according to the protocol established by Zhu et al.[13]. All data were recorded using a servohydraulic material testing machine (MTS 858 Bionix machine, MTS System Inc., Minneapolis, MN, USA). A pure moment of 2.5Nm was applied to the top vertebra (C1), while the specimen was allowed to move in an unconstrained 3-D manner. This continuous moment was applied at a rate of approximately 0.5°/s in all three primary directions of loading, namely flexion-extension, lateral bending, and axial rotation. The load was applied for five complete loading cycles. The first four cycles were used to precondition the specimen and minimize viscoelastic effects, and the fifth cycle was used for data analysis. The total angular range of motion was calculated for the last cycle. Throughout the fifth load cycle, inter segmental motions were collected via five infrared light-emitting diodes(LEDs)markers rigidly affixed to each of the C2 to C6 vertebrae, this serving as the definable points for three-dimensional motion. A marker carrier with four non-collinear LEDs on the base of the spine machine defined a general anatomical specimen coordinate system. A 3D laseroptoelectronic camera system (Optotrak 3020; Northern Digital, Waterloo, Canada; frame rate of 20 Hz) was used to measure the movement of the whole spine and of each segment. The kinematic behavior of each specimen was compared by examining the range of motion (ROM) of the C2-C4, C4-C5, C5-C6, and C2-C6 segments of intact and instrumented goat spines from the fifth loading cycle.

To determine the stability of the specimens in the different groups, the C6 and C7 segments were removed. A multidirectional flexibility test was used in a non-destructive manner for the C1-C5 segments. The end vertebrae of the specimens (C1 and C2, C4 and C5) were transfixed with perpendicular pins, to enhance the fixation with mounting jigs. The C1 to C5 vertebrae were then mounted in fast-drying epoxy resin (Huntsman Advanced Materials (HK) Limited, HK). A fatigue test comprised of 5,000 repetitions of axial rotation (fatigue load:1.0 Nm; frequency:0.25 Hz) was used during the kinematic test. A similar biomechanical test was performed. The kinematic behavior of each specimen was compared by examining the range of motion (ROM) and the neutral zone (NZ) of the C2-C4 segments of the intact and instrumented goat spines from the fifth loading cycle before and after the fatigue test. Throughout the biomechanical testing, special care was taken throughout the tests to keep the specimens moist using a 0.9% saline solution. A stability index ROM (SI ROM) and a stability index NZ (SI NZ) were introduced to quantify the stability provided by the implantation to the spine. These indices were similar to those described by Zhang et al. [14]. The SI ROM and SI NZ were defined using the following equations: SI ROM = (ROM_intact_ − ROM_instrumented_)/ROM_intact_, and SI NZ = (NZ_intact_ − NZ_instrumented_)/NZ_intact_.

### Histological evaluation

After the biomechanical testing, each sample was fixed in cold Karnovsky fixative containing 4% paraformaldehyde and 5% glutaraldehyde for two weeks. The specimens were dehydrated in an alcohol series and embedded in polymethyl-methacrylate. Transverse sections of 100 μm thickness were carried out with a Leica SP1600 sawing microtome (Leica, Nussloch, Germany). The specimens were then ground to the desired thickness. Masson’s trichrome staining and Van Gieson staining were used for transmitted light microscopy.

### Statistical analysis

All results were analyzed using SPSS Version 13.0 (SPSS Inc., Chicago, IL, USA). The data regarding surgery characteristics, ROM, NZ, and stiffness were analyzed using are repeated-measure analysis of variance (ANOVA). Post hoc tests were performed using Bonferroni correction for multiple comparisons. The level of statistical significance was set at P<0.05.

## Results

### Design of different artificial cervical joints

**Fig 2:**
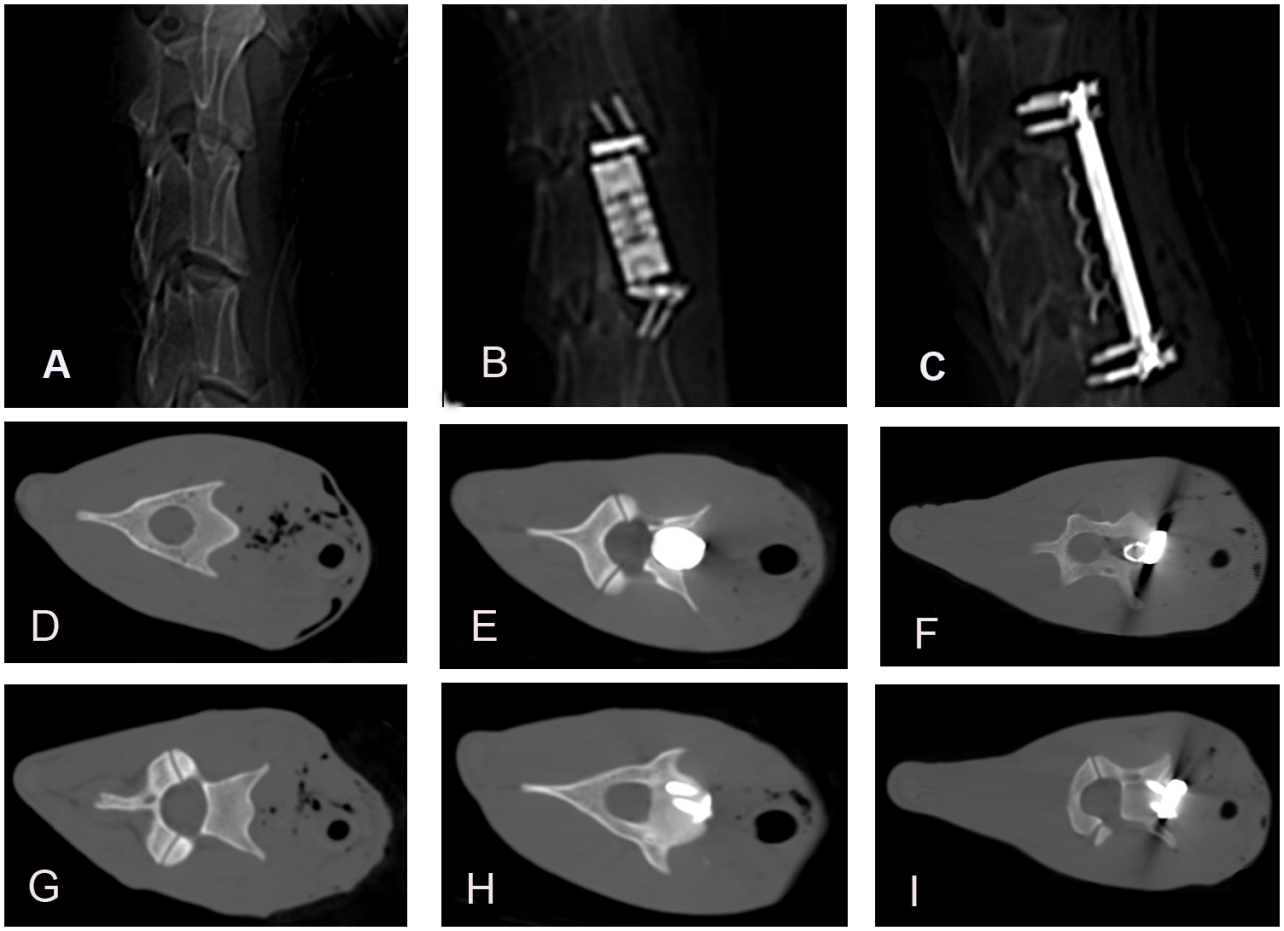
CT images of goats in control group, ACVC group, ACCF group 24 weeks after the surgery (ACVC and ACVC-HA appears similar). A, B, C, Lateral views of C2 to C4 for the three groups; D, G, transverse views of C3 and C4 vertebral bodies in control group; E, H, transverse views of C3 and C4 vertebral bodies in ACVC group; F, I, transverse views of C3 and C4 vertebral bodies in ACCF group.

**Fig 3:**
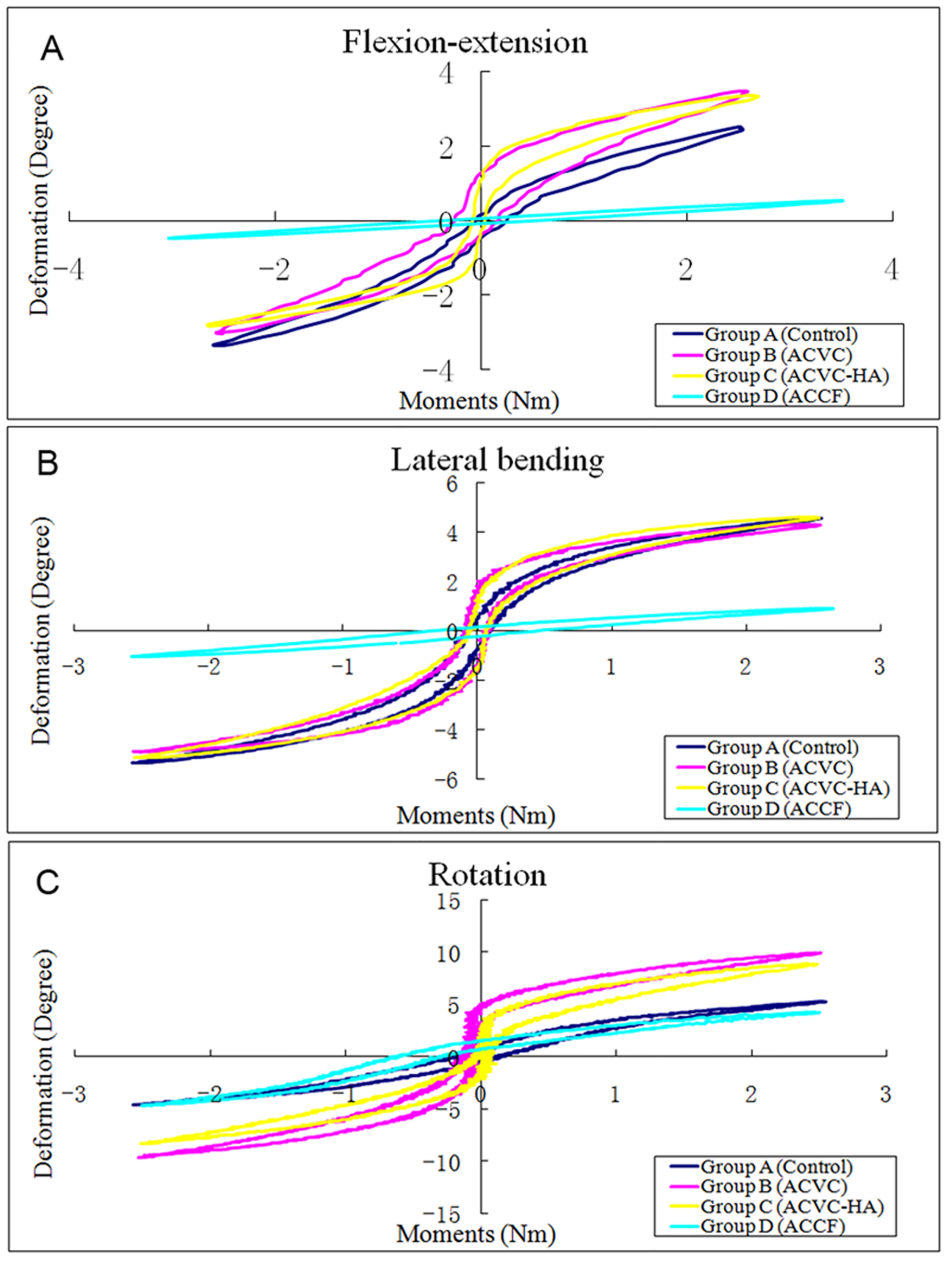
Typical hysteresis curves of C2-C4 level of the four groups before fatigue test in the directions of flexion-extension (A), lateral bending (B), and rotation (C).

**Fig 4:**
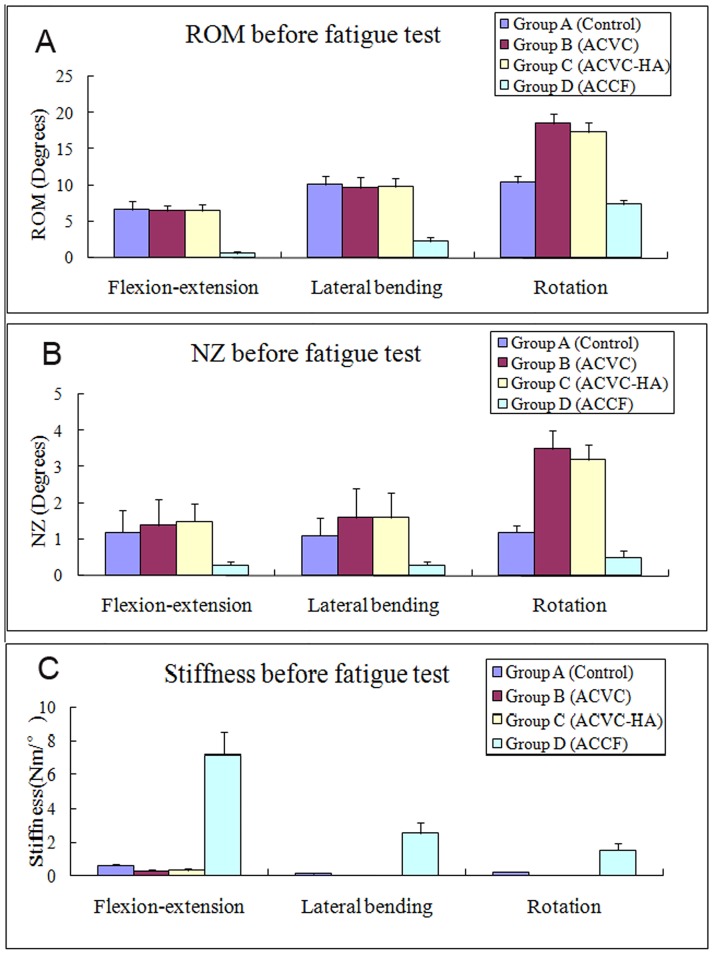
Average unidirectional ROM (A), NZ (B), and stiffness (C) of the four different groups at 24 weeks after surgery in all directions before the fatigue test.

**Fig 5:**
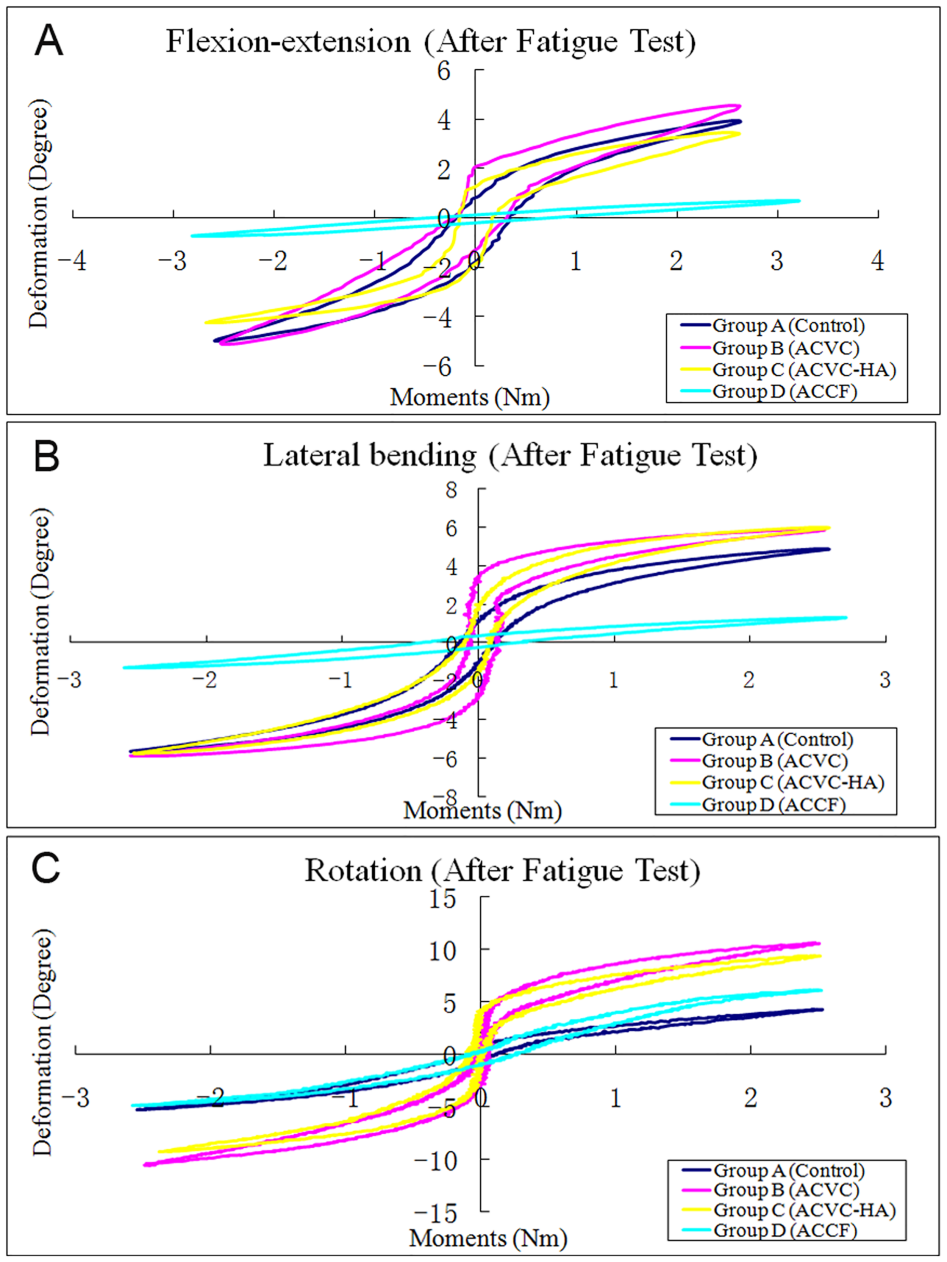
Typical hysteresis curves of C2-C4 level of the four groups after fatigue test in the directions of flexion-extension (A), lateral bending (B), and rotation (C).

**Fig 6:**
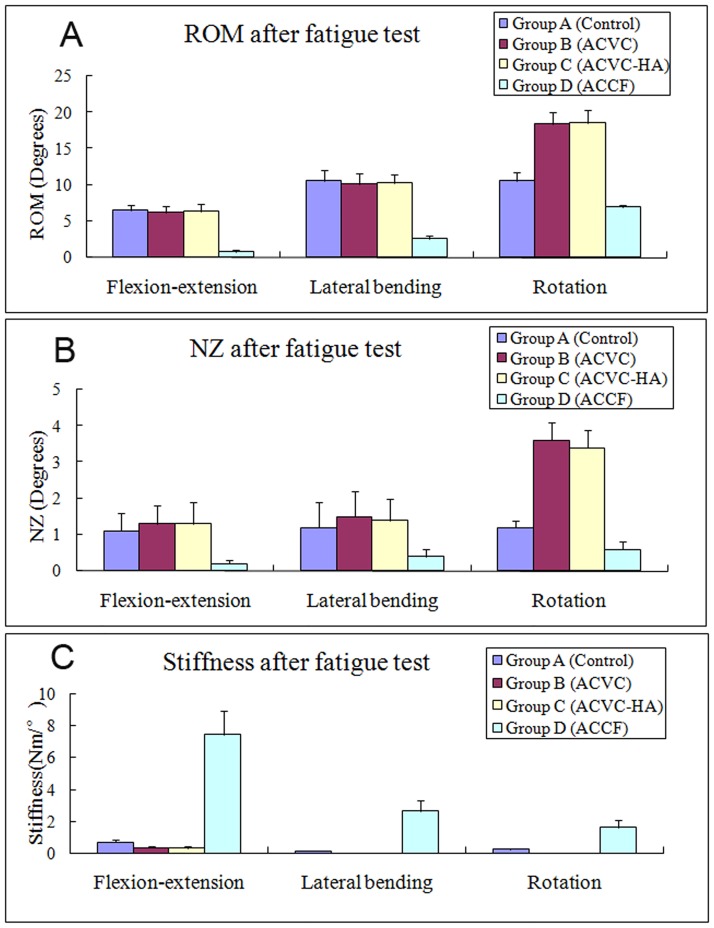
Average unidirectional ROM (A), NZ (B), and stiffness (C) of the four different groups at 24 weeks after surgery in all directions after the fatigue test.

**Fig 7:**
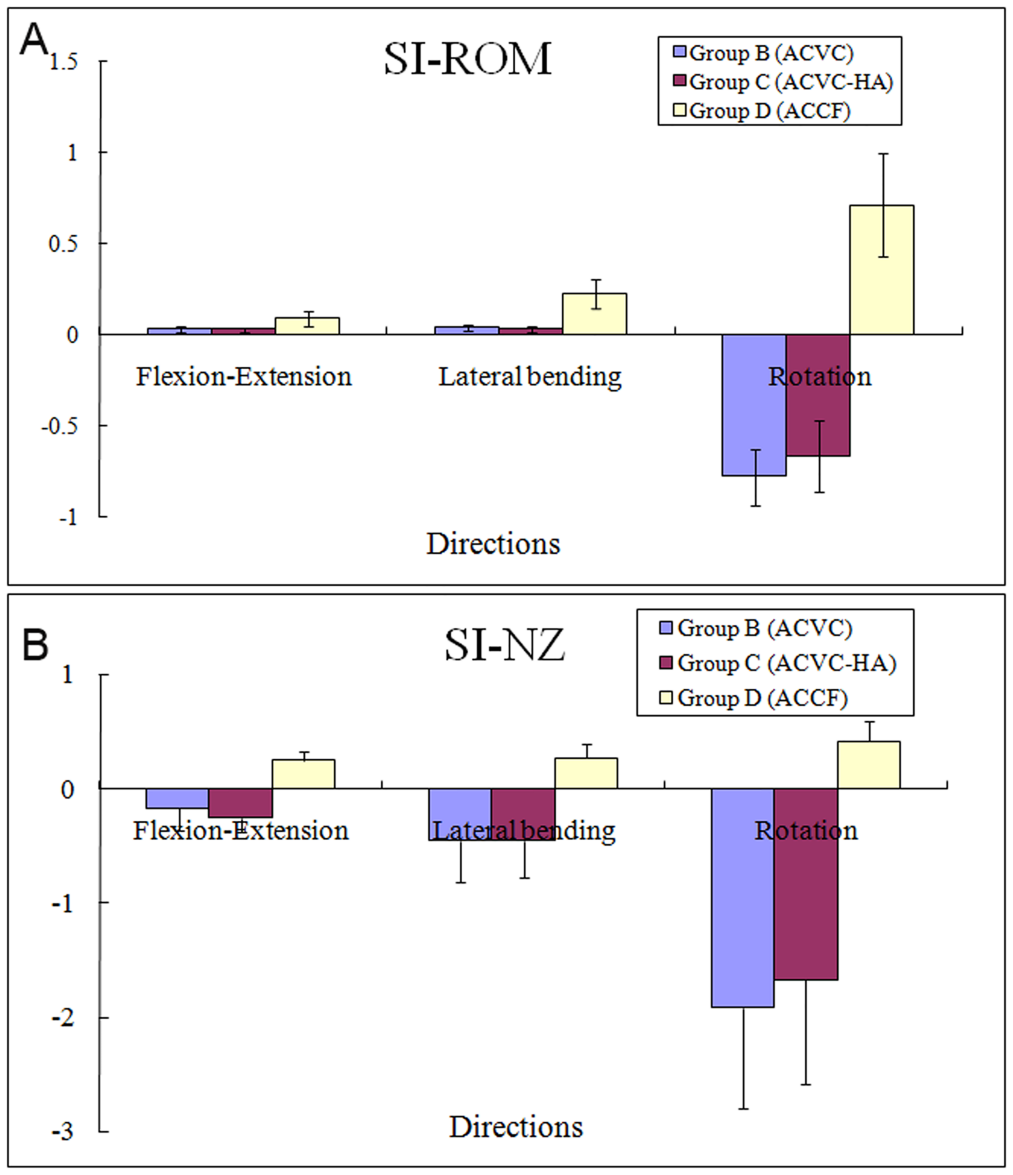
Average changes in the stability index ROMs and stability index NZs from ACVC group, ACVC-HA group and ACCF group.

**Fig 8:**
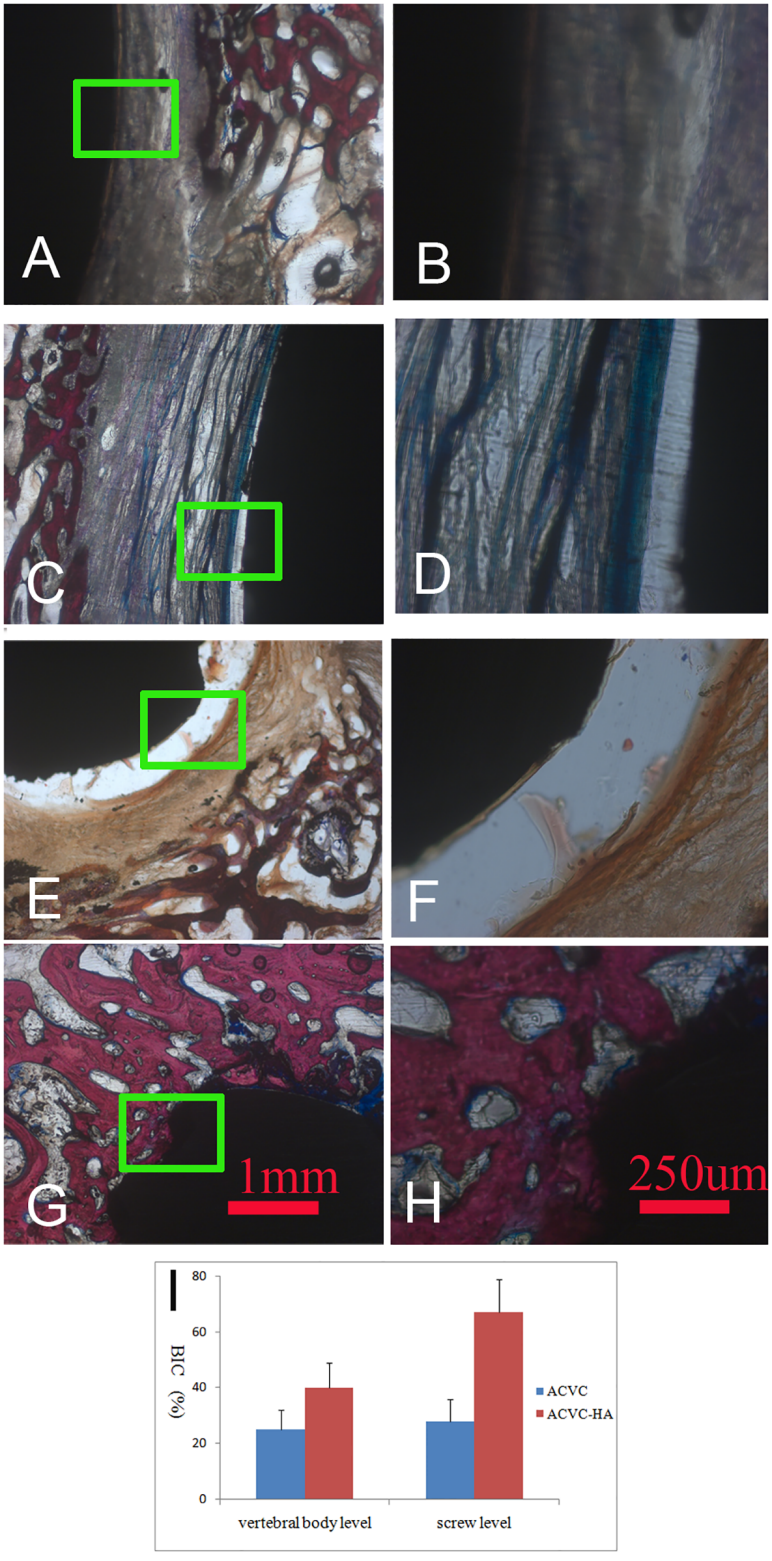
Comparison histological images of ACVC and ACVC-HA. A and B, 25X- and 100X- magnified images at the level of ACVC vertebral body component; C and D, 25X- and 100X- magnified images at the level of ACVC-HA vertebral body component; E and F, 25X- and 100X- magnified images at the level of fixation screws of ACVC; G and H, 25X- and 100X- magnified images at the level of fixation screws of ACVC-HA; I, Bone implant contact (BIC) of ACVC and ACVC-HA at vertebral body and screw level.

Fig 1 shows photographs of the ACVC (Fig 1B), the ACVC-HA (Fig 1C), and the titanium cage and the anterior plate (Fig 1D) implants. The ball-in-trough structure (Fig 1A-7 and 1A-8) is the most important part of this unconstrained metal-on-metal ACVC joint. In theory, the ball-in-trough articulation allows a 20° ROM in flexion-extension, a 12° ROM in lateral bending, a 360° ROM in rotation, and a 1.5-mm anterior-posterior slide horizontally. The ACVC-HA is an ACVC treated with a 30-μm hydroxyapatite biocoating via microarc oxidation technology. The titanium cage and the anterior plate were used for goat anterior cervical discectomy and fusion. The titanium cage was 8 mm wide and could be cut to a suitable length. Because the vertebral bodies of goats are much longer than those of humans, four to eight screws could be used to fix the plate and the cage. The vertebral components were hollow-structured, which allowed the surgeons to implant the autologous bone in the hollow space. Thus, the surrounding bone could grow into the vertebral components through the holes, this providing long-term stability.

### Clinical and radiological assessments of the different prosthesis implantations in goats

All surgeries were successful. Surgery time, blood loss, recovery time, and time before eating are shown in Table 1. No significant differences were found in surgery time, blood loss, recovery time, and time before eating among ACVC, ACVC-HA, and ACCF groups. These findings indicate that ACVC or ACVC-HA implantation did not increase surgery time, blood loss, recovery time, and time before eating compared with the ACCF group.

**Table 1:** Surgery characteristics of the four different groups.

	control group	ACVC group	ACVC-HA group	ACCF group
Surgery time (min)	12±6	68±11	67±16	65±13
Blood loss (ml)	10±6	55±18	56±19	51±20
Recovery time (hours)	1.1±0.6	3.6±1.7	4.1±2.0	3.5±1.4
Eating time (hours)	1.7±0.5	4.5±1.8	4.5±2.2	3.9±2.3

No significant difference was found among ACVC, ACVC-HA, and ACCF groups.

The goats tolerated the artificial joints very well during the experiment. All goats survived until 24 weeks after surgery. No abnormal neurological reflexes or neurological complications were found. One goat in the ACVC group and two goats in the ACCF group developed wound infections 8 to 12 days after surgery. These wound infections were cured by applying antibiotics and changing bandages daily. One goat in the ACVC-HA group and another goat in the ACCF group suffered respiratory infections and recovered by using antibiotics.

No bone fracture, joint dislocation, or screw loosening were found during the experimental period. Fig 2 shows the CT images of goats in the three different groups 24 weeks after surgery. In the ACVC group, the implant was properly located in the C3 vertebral body (Fig 2B). The vertebral body component of the ACVC was located in the middle of the C3 vertebral body (Fig 2E). The two lower screws were fixed to the C4 vertebral body (Fig 2H). The titanium cage was located between C2 and C4. The vertebral bodies from C2 to C4 were fixed by the plate (Fig 2C). The titanium cage was located in the trough of the C3 vertebral body (Fig 2F). The anterior plate was fixed by 3 screws to the C2 vertebral body and by 3 additional screws to the C4 vertebral body (Fig 2I).

### Biomechanical results of the different prosthesis implantations in goats

To investigate the cervical spine ROM separately, we used the three-dimensional laser optoelectronic camera system (Table 2). There were no significant differences among the control, ACVC, and ACVC-HA groups inflexion-extension and lateral bending for the C2-C6, C2-C4, C4-C5, and C5-C6 segments. The rotation ROM of the ACVC and ACVC-HA groups were greater than the ROM of the control group for the C2-C6 and C2-C4 segments. Accordingly, the ROM in rotation decreased in the ACVC and ACVC-HA groups for the C4-C5 and C5-C6 segments. For the C2-C6 and C2-C4 segments, the ROM was less for the ACCF group than for the control group in all three directions. Accordingly, the ROM of the C4-C5 segment in all three directions and the ROM of the C5-C6 segment in rotation increased in the ACCF group.

**Table 2:** Ranges of motion profiles of the cervical segments of the four groups before the fatigue test in all directions.

	Group A (Control group)	Group B (ACVC group)	Group C (ACVC-HA group)	Group D (ACCF group)
C2-4 (in Degrees)				
Flexion-Extension	4.0±0.6	3.9±0.6	4.0±0.3	0.4±0.1^a^,^b^
Lateral bending	5.9±0.7	5.6±0.8	5.8±0.5	1.3±0.3^a^,^b^
Rotation	7.2±0.6	11.2±0.9^a^	12.3±0.7^a^	5.6±0.5^a^,^b^
C4-5 (in Degrees)				
Flexion-Extension	1.3±0.2	1.5±0.3	1.4±0.3	2.2±0.3^a^,^b^
Lateral bending	1.8±0.2	2.0±0.3	2.0±0.4	2.9±0.3^a^,^b^
Rotation	2.2±0.3	1.2±0.3^a^	1.4±0.2^a^	3.4±0.2^a^,^b^
C5-6 (in Degrees)				
Flexion-Extension	1.4±0.2	1.3±0.2	1.4±0.1	1.6±0.2
Lateral bending	2.0±0.3	1.9±0.3	2.2±0.4	2.2±0.4
Rotation	2.4±0.2	1.8±0.2^a^	1.9±0.3^a^	1.6±0.1^a^,^b^
C2-6 (in Degrees)				
Flexion-Extension	6.5±0.9	6.2±0.7	6.3±1.1	4.2±0.4^a^,^b^
Lateral bending	9.6±1.1	9.1±1.5	9.2±0.9	6.9±0.4^a^,^b^
Rotation	11.2±0.8	14.5±1.3^a^	15.2±1.8^a^	9.8±0.8^a^,^b^

no significant differences between ACVC and ACVC-HA group

^a^ P<0.05 versus the Group A (control)

^b^ P<0.05 versus the Group B (ACVC group) or Group C (ACVC-HA group)

The biomechanical data of the C2-C4 segment recorded before the fatigue test (Table 3, Figs 3 and 4) confirmed that the ROM of ACVC and ACVC-HA groups in flexion-extension and lateral bending were similar to those of the control group. It also indicated that the ROM of ACVC and ACVC-HA groups in rotation increased compared with that of the control group. Also, compared with the control group, ROM in all directions was decreased in ACCF group.

**Table 3:** Ranges of motion, neutral zones, and stiffness profiles of the C2-4 segments of the four groups before the fatigue test in all directions.

Motion	Group A (Control group)	Group B (ACVC group)	Group C (ACVC-HA group)	Group D (ACCF group)
Range of Motion (in Degrees)				
Flexion-Extension	6.7±1.1	6.5±0.8	6.6±0.9	0.6±0.3^a^,^b^
Lateral bending	10.1±1.2	9.7±1.4	9.8±1.2	2.3±0.6^a^,^b^
Rotation	10.4±0.9	18.5±1.4^a^	17.3±1.3^a^	7.4±0.6^a^,^b^
Neural Zone (in Degrees)				
Flexion-Extension	1.2±0.6	1.4±0.7	1.5±0.5	0.3±0.1^a^,^b^
Lateral bending	1.1±0.5	1.6±0.8	1.6±0.7	0.3±0.1^a^,^b^
Rotation	1.2±0.2	3.5±0.5^a^	3.2±0.4^a^	0.5±0.2^a^,^b^
Stiffness (in Nm/Degree)				
Flexion-Extension	0.63±0.10	0.30±0.07^a^	0.33±0.11^a^	7.20±1.33^a^,^b^
Lateral bending	0.13±0.03	0.05±0.02^a^	0.05±0.01^a^	2.53±0.67^a^,^b^
Rotation	0.21±0.02	0.02±0.00^a^	0.02±0.00^a^	1.53±0.42^a^,^b^

no significant differences between ACVC and ACVC-HA group

^a^ P<0.05 versus the Group A (control)

^b^ P<0.05 versus the Group B (ACVC group) or Group C (ACVC-HA group)

There was no significant NZ difference in flexion-extension and lateral bending among control, ACVC and ACVC-HA groups. In rotation, NZ was significantly greater for ACVC and ACVC-HA groups than for the control group. For the ACCF group, NZ was significantly decreased in all directions. The stiffness was significantly decreased in all directions for the ACVC and ACVC-HA groups, while it was increased for the ACCF group. We found similar results for the ROM, NZ, and stiffness in the ACVC and ACVC-HA groups. Similar C2-C4 biomechanical data were found after the 5,000repetitions of the axial rotation fatigue test (Table 4, Figs 5 and 6).

**Table 4:** Ranges of motion, neutral zones, and stiffness profiles of the C2 to C4 segments of the four groups after the fatigue test in all directions.

Motion	Group A (Control group)	Group B (ACVC group)	Group C (ACVC-HA group)	Group D (ACCF group)
Range of Motion (in Degrees)				
Flexion-Extension	6.5±0.8	6.2±0.9	6.3±1.1	0.7±0.4^a^,^b^
Lateral bending	10.5±1.5	10.1±1.5	10.2±1.3	2.5±0.5^a^,^b^
Rotation	10.6±1.2	18.3±1.7^a^	18.5±1.9^a^	7.0±0.3^a^,^b^
Neural Zone (in Degrees)				
Flexion-Extension	1.1±0.5	1.3±0.5	1.3±0.6	0.2±0.1^a^,^b^
Lateral bending	1.2±0.7	1.5±0.7	1.4±0.6	0.4±0.2^a^,^b^
Rotation	1.2±0.3	3.6±0.5^a^	3.4±0.5^a^	0.6±0.2^a^,^b^
Stiffness (in Nm/Degree)				
Flexion-Extension	0.69±0.20	0.35±0.10^a^	0.34±0.12^a^	7.47±1.45^a^,^b^
Lateral bending	0.15±0.04	0.04±0.01^a^	0.05±0.02^a^	2.67±0.68^a^,^b^
Rotation	0.26±0.04	0.02±0.00^a^	0.02±0.00^a^	1.62±0.47^a^,^b^

no significant differences between ACVC and ACVC-HA group

^a^ P<0.05 versus the Group A (control)

^b^ P<0.05 versus the Group B (ACVC group) or Group C (ACVC-HA group)

The SI ROM and SI NZ are reported in Table 5 and Fig 7. The SI ROM of the ACVC and ACVC-HA groups were positive in flexion-extension and lateral bending but negative in rotation. The SI NZ of the ACVC and ACVC-HA group were negative in all directions. The SI ROM and SI NZ in the ACCF group were positive in all directions.

**Table 5:** Stability index of the flexibility test before the fatigue test.

Motion	Group B (ACVC group)	Group C (ACVC-HA group)	Group D (ACCF group)
Stability Index Range of Motion (in Degrees, SI ROM)			
Flexion-Extension	0.030±0.016	0.030±0.012	0.090±0.042*
Lateral bending	0.040±0.019	0.030±0.015	0.228±0.079*
Rotation	-0.779±0.156	-0.663±0.192	0.712±0.281*
Stability Index Neural Zone (in Degrees, SI NZ)			
Flexion-Extension	-0.167±0.198	-0.250±0.122	0.250±0.076*
Lateral bending	-0.4555±0.362	-0.455±0.321	0.273±0.122*
Rotation	-1.917±0.876	-1.667±0.911	0.417±0.182*

no significant differences between ACVC and ACVC-HA group

* P<0.05 versus the Group B (ACVC group) or Group C (ACVC-HA group)

### Histology

Histology sections of the goat cervical spines showed no inflammatory response 24 weeks after surgery, as well as no significant metallic wear debris at the implant-bone interface. Newly formed bone was found around the vertebral body component of the ACVC and the self-drilling fixation screws.

In the sections located at the ACVC vertebral body component level (Fig 8A and 8C), we found less new bone formation and more fibrous tissue around the implant for the ACVC group (Fig 8A) than for the ACVC-HA group (Fig 8C). In the sections located at the self-drilling fixation screw level (Fig 8E and 8G), we found significantly more new bone formation around the screws for the ACVC-HA group (Fig 8G) than for the ACVC group (Fig 8E). Moreover, the ACVC group had much more fibrous tissue around the screws than the ACVC-HA group. These findings indicate that, compared with the ACVC group, the ACVC-HA group had a significantly higher bone-implant contact (BIC) in both sections respectively located at the vertebral body component and screw level (Fig 8I), which is an appropriate index for measuring bone ingrowth into the implant.

The magnified images showed that bone formation around the implant surfaces was fragmentary and that a large portion of the bone tissue was separated from the implant surface by soft tissue in the ACVC group (Fig 8B and 8F). In contrast, in the ACVC-HA group, a large amount of the implant surface area was surrounded by a thin layer of cartilage on the vertebral body component of the ACVC (Fig 8D) and by extensive and continuous newly formed bone at the level of the fixation screws (Fig 8H).

## Discussion

ACCF and ACDF have been considered to represent the standard surgical treatments of cervical myelopathy or radiculopathy with degenerative disc disease that are refractory to conservative treatment. Because of its high rate of clinical success in relieving symptoms, ACCF is usually chosen to treat multi-level cervical degeneration disease[15]. The surgical treatments of multi-level cervical spine degeneration diseases by ACCF and ACDF seem to provide similar results. However, ACCF appears to provide a higher fusion rate than multi-segmental ACDF[16]. Although ACCF was considered to result in a higher fusion rate and a full exposure to complete decompression, the decreased number of fusion surfaces in multilevel ACDF leads to a lower risk of pseudarthrosis[17–19]. However, there is currently no powerful evidence for the superiority of one method over the other. Weber et al.[20] performed a retrospective cohort study to assess the subsidence and revision rates associated with different interbody cages following anterior cervical corpectomy and reconstruction. They found that for the fibula allograft, the average graft subsidence was 3 mm with are vision rate of 25% while these were 2.9 mm and 11.1%, and 2.9 mm and 18.8% respectively for the titanium mesh cages and the titanium expandable cages, indicating that the titanium cage did not increase the subsidence after cervical fusion. Therefore, we chose ACCF using titanium cages and anterior cervical plates as the gold standard to compare the clinical and biomechanical properties of the ACVC and ACVC-HA.

### Mobility and long-term stability are essential for ACVC

The two main advantages of this metal-on-metal unconstrained artificial cervical joint system are its ability to allow physiological motions and its potential to reduce stress at the bone-implant interface. This articulation allows anterior-posterior translation, constrained flexion-extension, and unconstrained rotation, which theoretically mimics the normal cervical physiological motion. To eliminate the risk of dislocation, it is important to have the articulation closed and the posterior longitudinal ligament and surrounding soft tissues aid in providing stability.

Besides, we designed several structures to enhance the long-term stability, such as the HA biocoating, the rough and grit-blasted endplate component, and the hollow vertebral body component. Autologous grafting is the gold criteria for fusion in various types of ACCF techniques because it has a high fusion rate compared with discectomy alone or implanted cages. However, the main shortcoming of autologous bone graft is the harvesting of bone, primarily from the iliac crest, which may results in short- and long-term morbidities. These include increased blood loss, infection, donor site pain, hematoma, and peripheral nerve injury[21]. Compared with autografts, allografts have a lower fusion rate, as well as increased infection and immune issues. In contrast, interbody cages provide initial stability and require much less structural bone graft. The anterior plate can provide immediate stability and maintain the cervical spine alignment. This is the reason why we designed a hollow structure to hold autologous bone from the corpectomy, in order to exclude the donor site pain and promote bone ingrowth.

The selection of biomaterials for the artificial joint system depend on the good mechanical properties (high strength and low modulus closer to bone), biocompatibility, superior corrosion and wear resistance, and osseointegration. The reasons we chose Ti6Al4V were because of its adequate mechanical properties, good biocompatibility, valuable imaging characteristics, and low elasticity modulus. Besides, there was no acute neural orsystemic histopathologic response to the Ti6Al4V included in a 6-month in vivo animal model[22]. But long term performance of these alloys has raised some concerns of release of aluminum and vanadium ions, poor shear strength, severe wear, which may result in inflammatory reaction leading to loosening of implants due to osteolysis.

### ACVC and ACVC-HA preserve cervical motion compared with ACCF

We designed this ACVC implant to preserve motion in the degenerative segment of the cervical spine after the anterior cervical discectomy and subtotal corpectomy. To test the ACVC biomechanical properties, we compared its kinematic data with those of the intact and ACCF states.

First of all, we investigated the adjacent segmental movement. Our data confirmed that once the cervical vertebrae (C2-C4) were fused, the adjacent segment (C4-C5) would compensate for the decreased ROM. Finally, the total motion of the cervical spine (C2-C6) was still less than that of the control group. In contrast, by using this novel motion-preserving cervical joint system, namely ACVC or ACVC-HA, the movement of C2-C4 could be preserved and C4-C5 would not compensate for the altered ROM in flexion-extension and lateral bending. Moreover, rotation increased at the C2-C4 level after ACVC and ACVC-HA implantations, which resulted in a decreased ROM for the C4-C5 and C5-C6 segments. Park et al.[23] reported that the segmental rotation, translation, center of rotation, disc height, and disc angle were increased after arthroplasty, while they were unaltered after fusion in an in vivo kinematic study. Prasarn et al.[24] also showed that the biomechanics was changed at the levels adjacent to a cervical spine fusion, notably the adjacent segmental motion which was increased. Schwab et al. [25]reported that motion compensation increases at segments immediately adjacent to a single-level fusion. Our data showed that the closest segment played an important role in compensating for the loss of cervical mobility, which indicated that the ACVC maintained a physiological distribution of ROM throughout the cervical spine.

Compared with the intact state (control group), no difference was observed for both ACVC and ACVC-HA groups in the ROM and NZ during flexion-extension and lateral bending. During rotation, the ROM and NZ were increased. The stiffness was decreased in all directions. The fold change of stiffness decrease in rotation (0.10) was much greater than that in flexion-extension (0.48) and lateral bending (0.38). Compared with the ACCF group, the ROM and NZ were significantly increased and the stiffness decreased in all directions. These biomechanical results indicated that thanks to the ball-in-trough articulation structure design, the ACVC could preserve the three-dimensional motion of cervical spine. Compared with the intact state, the fold change of ROM decrease in the ACCF group was much greater in flexion-extension (0.09) than in lateral bending (0.23) and rotation (0.71). The fold change of NZ decrease was also much greater in flexion-extension (0.25) than in lateral bending (0.27) and rotation (0.42). The fold change of increase in stiffness was much greater in lateral bending (×19.46) than in flexion-extension (×11.43) and rotation (×7.29). The cylinder titanium cage combined with the anterior plate successfully provided strong stability in all directions. A positive, null, or negative stability index value indicates that the spinal construct was respectively, more stable than, equally as stable as, or less stable than the intact spine. Therefore, a higher stability index value indicates a greater immediate stability of the spinal construct [14]. In the present study, the SI ROM and negative SI NZ values observed in the ACVC group were expected because of the ball-in-trough and anterior-posterior slide design of the articulation. The SI ROM and SI NZ values observed in the ACCF group confirmed that this method can provide excellent stability after discectomy and corpectomy.

All of the above results indicate that, compared with the intact state and the ACCF group, the ACVC (or ACVC-HA) successfully restored the flexion-extension and lateral bending abilities after discectomy and subtotal corpectomy. The absence of any biomechanical difference before and after the fatigue test for every group indicate that the fixation screw could provide primary stability.

### The HA coating could enhance the long-term stability by improved BIC

The histological results indicated that the amount of new bone formed was much greater in the ACVC-HA than the ACVC group. Furthermore, the bone-implant contact was much higher in the ACVC-HA than the ACVC group. This higher bone-implant contact indicate that the HA biocoating offered great osteoconductivity.

In the sections located at the screw level, we found fibrous tissue around the screws in the ACVC group and newly formed bone in the ACVC-HA group. This may have occurred because there were less micro-motion and osteolysis after implantation in the ACVC-HA than the ACVC group. The HA coating formed a strong interface between the bone and screws, providing strong initial stability in the early stage after implantation. The MAO-treated, HA-coated screws significantly improved the bone reaction to implant and increased the fixation stability. Thus, they served as an effective method to decrease screw-loosening by providing both mechanical interlocking and biochemical support at the bone-implant interface.

The histological results suggest that the HA coating may encourage bony ingrowth to provide a morphological fixation of the implant to the bone, which may improve the interlocking between the implant and the bone. The bony ingrowth into the implant is accelerated by the bioactivity and the osteoconductive property of the HA coating. The HA coating on the titanium alloy implant increased the osteoconductivity and inhibited soft tissue infiltration, thus offering a mechanical interlocking attachment for short- and long-term fixation. Furthermore, the HA coating immobilizes osteoblastic cells and facilitate vascularization in bone tissue at the early stage after implantation. During the later stage, peri-implant bone formation was stimulated by the calcium ions degraded by the HA coating [26].

Our histological results are in agreement with the findings of other studies that have used calcium-phosphate ceramics for various applications. Lim et al.[27] performed an in vitro pilot study and found that microarc oxidation could be a reasonable option for treating titanium alloy surfaces in order to facilitate a better osseointegration. Huang et al.[28] reported that the apatite layer on the titanium implant processed with microarc oxidation is expected to enhance both the bony ingrowth into the implant and the interlocking between the implant and bone. Quaranta et al. [29]reported that hydroxyapatite coatings have an obvious advantage regarding osteogenesis facilitation and play a critical role in the stability of prostheses. Our results are consistent with those of other studies reporting that HA has a stimulatory effect on bone osseointegration and osteogenesis.

### Research limitations

Several limitations are inherent to this study. First of all, this is an in vivo animal study. The length and size of cervical vertebrae in goat is different from those of humans. Besides, in humans, the most common symptomatic levels are C5/6 and C6/7. Agenetic and environmental etiology may likely explain this difference. In our study, we choose the C2-C4 level as the surgery level, because of the biomechanical properties of C2-C4 in goat, which is similar to those of humans. Secondly, the follow-up duration is limited. In animals, it may be appropriate to follow an in vivo study up to only 24 weeks. However, it is difficult to comment on the function and longevity of a device designed to remain intact and preserve motion after only 24 weeks in humans. That is why we included a fatigue protocol in the biomechanical test, though the number of fatigue cycles was limited as well. Thirdly, we are planning to conduct further experiment aiming at improving the durability and stabilization capacity of our ACVC for clinical application, such as applying biocoating on the surface of ACVC to improve the bone-implant integration for long-term stability. Fourthly, the low wear resistance of Ti6Al4V was the main shortcoming of this joint system, which may result in implant loosening and wear debris. Thus we are planning to develop a metal-on-UHMWPE articulation to solve this problem.

## Conclusions

We have successfully performed the in vivo implantation of ACVC, ACVC-HA and titanium cage in goats. Goat has been proven to been excellent animal model for cervical spine biomechanical studies, especially for the artificial joints and biomedical materials. The biomechanical results indicate that, compared with intact state and ACCF, ACVC and ACVC-HA provide stability and preserve segmental movement for at least 6 months after discetcomy and corpectomy. The histological results demonstrate that hydroxyapatite biocoating provide better bone ingrowth, which is essential for long-term stability. Also, ACVC-HA brings a new insight to treat cervical degenerative disease.

## References

[1] Rao RD, Currier BL, Albert TJ, Bono CM, Marawar SV, Poelstra KA, et al Degenerative cervical spondylosis: clinical syndromes, pathogenesis, and management. J Bone Joint Surg Am. 2007;89(6):1360–78. . 17575617 10.2106/00004623-200706000-00026

[2] Shen FH, Samartzis D, Khanna N, Goldberg EJ, An HS. Comparison of clinical and radiographic outcome in instrumented anterior cervical discectomy and fusion with or without direct uncovertebral joint decompression. Spine J. 2004;4(6):629–35. Epub 2004/11/16. 10.1016/j.spinee.2004.04.009 . 15541694

[3] Papadopoulos EC, Huang RC, Girardi FP, Synnott K, Cammisa FP Jr. Three-level anterior cervical discectomy and fusion with plate fixation: radiographic and clinical results. Spine (Phila Pa 1976). 2006;31(8):897–902. Epub 2006/04/20. 10.1097/01.brs.0000209348.17377.be . 16622378

[4] Cho SK, Riew KD. Adjacent segment disease following cervical spine surgery. J Am Acad Orthop Surg. 2013;21(1):3–11. 10.5435/JAAOS-21-01-3 . 23281466

[5] Goffin J, Geusens E, Vantomme N, Quintens E, Waerzeggers Y, Depreitere B, et al Long-term follow-up after interbody fusion of the cervical spine. Journal of spinal disorders & techniques. 2004;17(2):79–85. Epub 2004/07/21. . 15260088 10.1097/00024720-200404000-00001

[6] Matsumoto M, Okada E, Ichihara D, Watanabe K, Chiba K, Toyama Y, et al Anterior cervical decompression and fusion accelerates adjacent segment degeneration: comparison with asymptomatic volunteers in a ten-year magnetic resonance imaging follow-up study. Spine (Phila Pa 1976). 2010;35(1):36–43. 10.1097/BRS.0b013e3181b8a80d . 20023606

[7] Dmitriev AE, Cunningham BW, Hu N, Sell G, Vigna F, McAfee PC. Adjacent level intradiscal pressure and segmental kinematics following a cervical total disc arthroplasty: an in vitro human cadaveric model. Spine (Phila Pa 1976). 2005;30(10):1165–72. Epub 2005/05/18. . 15897831 10.1097/01.brs.0000162441.23824.95

[8] Cepoiu-Martin M, Faris P, Lorenzetti D, Prefontaine E, Noseworthy T, Sutherland L. Artificial cervical disc arthroplasty: a systematic review. Spine (Phila Pa 1976). 2011;36(25):E1623–33. Epub 2011/11/22. 10.1097/BRS.0b013e3182163814 . 22101705

[9] Eliaz N, Ritman-Hertz O, Aronov D, Weinberg E, Shenhar Y, Rosenman G, et al The effect of surface treatments on the adhesion of electrochemically deposited hydroxyapatite coating to titanium and on its interaction with cells and bacteria. Journal of materials science Materials in medicine. 2011;22(7):1741–52. 10.1007/s10856-011-4355-y . 21611792

[10] Xie J, Luan BL. Microstructural and electrochemical characterization of hydroxyapatite-coated Ti6Al4V alloy for medical implants. Journal of Materials Research. 2008;23(3):768–79.

[11] Qin J, He X, Wang D, Qi P, Guo L, Huang S, et al Artificial cervical vertebra and intervertebral complex replacement through the anterior approach in animal model: a biomechanical and in vivo evaluation of a successful goat model. PloS one. 2012;7(12):e52910 Epub 2013/01/10. 10.1371/journal.pone.0052910 ; 23300816 PMC3531380

[12] Rocca M, Orienti L, Stea S, Moroni A, Fini M, Giardino R. Comparison among three different biocoatings for orthopaedic prostheses. An experimental animal study. Int J Artif Organs. 1998;21(9):553–8. Epub 1998/11/25. . 9828062

[13] Zhu Q, Larson CR, Sjovold SG, Rosler DM, Keynan O, Wilson DR, et al Biomechanical evaluation of the Total Facet Arthroplasty System: 3-dimensional kinematics. Spine (Phila Pa 1976). 2007;32(1):55–62. Epub 2007/01/05. 10.1097/01.brs.0000250983.91339.9f . 17202893

[14] Zhang J, He X, Li H, Wang D, Zhao W, Xu J, et al Biomechanical study of anterior cervical corpectomy and step-cut grafting with bioabsorbable screws fixation in cadaveric cervical spine model. Spine (Phila Pa 1976). 2006;31(19):2195–201. Epub 2006/09/02. 10.1097/01.brs.0000232798.97075.73 . 16946653

[15] Jiang SD, Jiang LS, Dai LY. Anterior cervical discectomy and fusion versus anterior cervical corpectomy and fusion for multilevel cervical spondylosis: a systematic review. Arch Orthop Trauma Surg. 2012;132(2):155–61. 10.1007/s00402-011-1402-6 . 21968573

[16] Mummaneni PV, Kaiser MG, Matz PG, Anderson PA, Groff MW, Heary RF, et al Cervical surgical techniques for the treatment of cervical spondylotic myelopathy. Journal of neurosurgery Spine. 2009;11(2):130–41. 10.3171/2009.3.SPINE08728 . 19769492

[17] Rao RD, Gourab K, David KS. Operative treatment of cervical spondylotic myelopathy. J Bone Joint Surg Am. 2006;88(7):1619–40. 10.2106/JBJS.F.00014 . 16818991

[18] Song KJ, Lee KB, Song JH. Efficacy of multilevel anterior cervical discectomy and fusion versus corpectomy and fusion for multilevel cervical spondylotic myelopathy: a minimum 5-year follow-up study. Eur Spine J. 2012;21(8):1551–7. 10.1007/s00586-012-2296-x ; 22526699 PMC3535261

[19] Yalamanchili PK, Vives MJ, Chaudhary SB. Cervical spondylotic myelopathy: factors in choosing the surgical approach. Advances in orthopedics. 2012;2012:783762 10.1155/2012/783762 ; 22312563 PMC3270546

[20] Weber MH, Fortin M, Shen J, Tay B, Hu SS, Berven S, et al Graft Subsidence and Revision Rates Following Anterior Cervical Corpectomy: A Clinical Study Comparing Different Interbody Cages. Clinical spine surgery. 2016 10.1097/BSD.0000000000000428 . 27623304

[21] McConnell JR, Freeman BJ, Debnath UK, Grevitt MP, Prince HG, Webb JK. A prospective randomized comparison of coralline hydroxyapatite with autograft in cervical interbody fusion. Spine (Phila Pa 1976). 2003;28(4):317–23. Epub 2003/02/19. 10.1097/01.BRS.0000048503.51956.E1 . 12590203

[22] Cunningham BW. Basic scientific considerations in total disc arthroplasty. Spine J. 2004;4(6 Suppl):219S–30S. 10.1016/j.spinee.2004.07.015 . 15541670

[23] Park DK, Lin EL, Phillips FM. Index and adjacent level kinematics after cervical disc replacement and anterior fusion: in vivo quantitative radiographic analysis. Spine (Phila Pa 1976). 2011;36(9):721–30. 10.1097/BRS.0b013e3181df10fc . 20543765

[24] Prasarn ML, Baria D, Milne E, Latta L, Sukovich W. Adjacent-level biomechanics after single versus multilevel cervical spine fusion. Journal of neurosurgery Spine. 2012;16(2):172–7. 10.3171/2011.10.SPINE11116 . 22136389

[25] Schwab JS, Diangelo DJ, Foley KT. Motion compensation associated with single-level cervical fusion: where does the lost motion go? Spine (Phila Pa 1976). 2006;31(21):2439–48. 10.1097/01.brs.0000239125.54761.23 . 17023853

[26] Fielding GA, Roy M, Bandyopadhyay A, Bose S. Antibacterial and biological characteristics of silver containing and strontium doped plasma sprayed hydroxyapatite coatings. Acta biomaterialia. 2012;8(8):3144–52. 10.1016/j.actbio.2012.04.004 ; 22487928 PMC3393112

[27] Lim YW, Kwon SY, Sun DH, Kim HE, Kim YS. Enhanced cell integration to titanium alloy by surface treatment with microarc oxidation: a pilot study. Clin Orthop Relat Res. 2009;467(9):2251–8. Epub 2009/05/13. 10.1007/s11999-009-0879-6 ; 19434468 PMC2866922

[28] Huang P, Zhang Y, Xu K, Han Y. Surface modification of titanium implant by microarc oxidation and hydrothermal treatment. J Biomed Mater Res B Appl Biomater. 2004;70(2):187–90. Epub 2004/07/21. 10.1002/jbm.b.30009 . 15264299

[29] Quaranta A, Iezzi G, Scarano A, Coelho PG, Vozza I, Marincola M, et al A histomorphometric study of nanothickness and plasma-sprayed calcium-phosphorous-coated implant surfaces in rabbit bone. J Periodontol. 2010;81(4):556–61. 10.1902/jop.2010.090296 . 20367097

